# The Occurrence of Warfarin-Related Nephropathy and Effects on Renal and Patient Outcomes in Korean Patients

**DOI:** 10.1371/journal.pone.0057661

**Published:** 2013-04-01

**Authors:** Jung Nam An, Shin Young Ahn, Chang-Hwan Yoon, Tae-Jin Youn, Moon-Ku Han, Sejoong Kim, Ho Jun Chin, Ki Young Na, Dong-Wan Chae

**Affiliations:** 1 Department of Internal Medicine, Seoul National University Hospital, Seoul National University College of Medicine, Seoul, Korea; 2 Division of Nephrology, Department of Internal Medicine, Seoul National University Bundang Hospital, Seongnam-si, Gyeonggi-do, Korea; 3 Division of Cardiology, Department of Internal Medicine, Seoul National University Bundang Hospital, Seongnam-si, Gyeonggi-do, Korea; 4 Department of Neurology Stroke Center, Neuroscience Center, Seoul National University Bundang Hospital, Seongnam-si, Gyeonggi-do, Korea; University of Sao Paulo Medical School, Brazil

## Abstract

**Background:**

Warfarin-related nephropathy (WRN) is a recently described disease entity, in which excessive warfarinization (international normalized ratio (INR) >3.0) causes acute kidney injury. Previous reports regarding WRN included few Asian patients who might have differed from the western WRN patients in terms of genetic and environmental factors.

**Methods:**

During the period of March 2003 to December 2011, the data about a total of 1297 patients who had serum creatinine (sCr) level measured within 1 week after INR >3.0 and within 6 months before INR >3.0 was analyzed through the retrospective review of electronic medical records of a single tertiary hospital in Korea.

**Result:**

WRN developed in 19.3% of patients having excessive warfarinization. The incidence was higher in the chronic kidney disease (CKD) group than the non-CKD group. The risk of WRN increased as the basal serum albumin level decreased and was strongly associated with highest quartile serum AST level at post INR elevation and the presence of congestive heart failure. But the presence of atrial fibrillation was protective against the development of WRN. Neither the presence of CKD nor basal estimated glomerular filtration rate (eGFR) was an independent risk factor for WRN. Despite no difference in the basal sCr level, the sCr level was higher in patients with WRN than those without WRN after follow-up. The mortality rates were also higher in patients with WRN.

**Conclusions:**

WRN developed in 19.3% of patients having excessive warfarinization. A lower basal serum albumin, highest quartile serum AST level at post INR elevation, and congestive heart failure were associated with the occurrence of WRN. The development of WRN adversely affected renal and patient outcomes.

## Introduction

Warfarin, the most commonly prescribed oral anticoagulant, interrupts the synthesis of coagulation factors (II, VII, IX, and X) by inhibiting the C1 subunit of the vitamin K epoxide reductase enzyme complex and causes disruption of the extrinsic clotting cascade [1], [2].

Warfarin-related nephropathy (WRN) is a recently described disease entity, in which excessive warfarinization [international normalized ratio (INR) >3.0] causes acute kidney injury without the evidence of clinically relevant hemorrhage [3]. Glomerular hemorrhage and tubular obstruction by red blood cell casts were reported to be a major mechanism of acute kidney injury (AKI) associated with WRN [4], and a structurally abnormal glomerular basement membrane was also related to the increased risk for glomerular hemorrhage [5].

Although WRN was originally described in patients who had already had chronic kidney disease (CKD) [4], [6], this complication of warfarin commonly developed in patients without CKD, albeit less frequently, as well as in patients with CKD. The occurrence of WRN adversely affected renal and patient outcomes in patients with and without CKD [3].

Warfarin is metabolized and removed primarily in the liver through the cytochrome P450 pathway. Warfarin has a narrow therapeutic range for anticoagulation and has great differences in individual dose requirements. The fact that a multitude of different environmental factors, including diet and drugs, and genetics can affect the pharmacokinetics and pharmacodynamics of warfarin [7], [8] suggests the need to perform studies on WRN in different races or countries. No studies related to WRN in Asian patients have yet been reported, however.

Therefore, we aimed to investigate and analyze the incidence, clinical features, risk factors, and prognosis, including mortality rate, of presumed WRN in Korean people by retrospective analysis of the electric medical records of a single tertiary hospital in Korea.

## Subjects and Methods

### Study population

During the period of March 2003 to December 2011, a total of 1425 warfarin-treated patients over 18 years of age who had at least one event of INR >3.0 and also had serum creatinine (sCr) level measured within 1 week after INR >3.0 and within 6 months before INR >3.0 were identified in Seoul National University Bundang Hospital. In cases with multiple events of INR >3.0, the first event was used for analysis. After the exclusion of patients with end-stage renal disease maintained on renal replacement therapy, and patients with unreliably high basal estimated glomerular filtration rate (eGFR) more than 175 ml/min/1.73 m^2^, a total of 1297 patients were enrolled in this retrospective study. This study was approved by the Seoul National University Bundang Hospital Institutional Review Board, and the need for informed consent from the patients was waived because of its retrospective design. All clinical investigations were conducted in accordance with the guidelines of the 2008 Declaration of Helsinki.

### Data collection

Demographics and baseline clinical characteristics, including the medical history, co-morbid diseases, and indications for warfarin therapy, were assessed from the initiation of warfarin therapy to the event of INR >3.0 by examination of the electronic medical records.

Overall co-morbid diseases were defined by diagnosis codes based on International Statistical Classification of Diseases and Related Health Problems, 10^th^ revision (ICD-10). Hypertension, diabetes mellitus, and thyroid disease were also defined as concurrent use of antihypertensive drugs, oral hypoglycemic agents or insulin, and thyroid hormone or thyroid-suppressant drugs, respectively. Congestive heart failure (CHF) or coronary and peripheral artery disease was also defined by echocardiography or coronary and peripheral artery angiography. Respiratory disease included tuberculosis, chronic obstructive pulmonary disease, asthma, and interstitial lung disease. The indications for warfarin therapy were classified into five entities as follows: atrial fibrillation, cerebrovascular attack, venous thrombosis, arterial embolism, and valve diseases.

The laboratory findings that could influence and be associated with the development of WRN [INR, sCr, eGFR, hemoglobin (Hb), hematocrit (Hct), platelet count, serum calcium, phosphorus, cholesterol, total protein, albumin, and total bilirubin level] were obtained from the electronic medical records. Baseline laboratory findings, which were defined as the latest measurements within 6 months before INR >3.0 and laboratory findings at the event of INR >3.0, were collected. In our hospital, sCr had been measured by Jaffe method until September 7, 2009 and since then sCr has been measured by the assay based on isotope dilution mass spectrometry (IDMS). So, we substituted sCr (by IDMS) for sCr (by Jaffe method) using following equation: sCr (by IDMS)  = 1.1081×sCr (by Jaffe method) –0.3109. Estimated GFR was calculated using the following IDMS-traceable Modification of Diet in Renal Disease (MDRD) equation: GFR (ml/min/1.73 m^2^)  = 175× (Scr^)−1.154^× (age in years)^−0.203^× (0.742 if female).

In this study, CKD was defined as eGFR <60 ml/min/1.73 m^2^, regardless of proteinuria or microalbuminuria, largely because proteinuria and other parameters suggestive of renal damage were not available on all recruited patients.

WRN was defined as more than 50% increase or more than 0.3 mg/dL elevation of the sCr level measured within 1 week after INR >3.0 over the baseline sCr level measured within 6 months before INR >3.0, based on the Acute Kidney Injury Network (AKIN) criteria published in 2007 [9]. Mortality data, including the date of death and the cause of death, was obtained and collected from the Statistics Korea (KOSTAT).

### Statistical analysis

Categorical variables described as frequency and proportion, were compared with the chi-square test; continuous variables expressed as the mean ± standard deviation were compared by the Student's *t-*test. The laboratory findings, including INR, sCr, serum calcium, phosphorus, protein, albumin, cholesterol, alkaline phosphatase, AST, and ALT levels, were divided into quartiles for analysis. Cox proportional hazard regression models were used to examine the association between multiple risk factors and the development of WRN, and determine the unadjusted odds ratios (OR) and 95% confidence intervals (CI). In multivariate analysis, statistically significant covariates from the univariate analysis (p<0.1) and clinically important covariates were selected. The incidence of WRN and the mortality rate according to the presence of WRN were evaluated by Kaplan-Meier analysis (log-rank test). A p-value of less than 0.05 was considered statistically significant. Statistical analysis was performed with SPSS version 18.0 K software (SPSS Inc., Chicago, IL, USA).

## Results

### The incidence of warfarin-related nephropathy

Among 1297 recruited patients, 670 (51.7%) were male, and the mean age was 68.4±12.5 years. The mean duration of follow-up in this study was 23.3±26.8 (range 0.04 to 106.10) months, and the mean period from the administration of warfarin to the event of INR >3.0 was 8.5±17.1 (range 0.04 to 104.33) months. During the follow-up period, WRN developed in 250 patients, constituting 19.3% of all recruited patients. The remaining 1047 (80.7%) patients had the event of INR >3.0 but not enough of an increase in the sCr level to satisfy AKIN criteria. The majority of cases of WRN (82.2%) occurred within the first year after the initiation of warfarin therapy. WRN developed in 24.0% of patients with CKD and in 17.4% of patients without CKD (Table 1).

**Table 1:** Demographic and clinical baseline characteristics of patients with and without WRN.

	No WRN (N = 1047, 80.7%)	WRN (N = 250, 19.3%)	Total (N = 1297)	*P*-value
**Male**	544 (52.0)	126 (50.4)	670 (51.7)	0.658
**Age** *	68.1±12.7	69.6±11.8	68.4±12.5	0.093
**Duration** * **(WFR-INR >3.0)** †	8.6±17.0	8.0±17.5	8.5±17.1	0.619
**Duration** * **(WFR-F/U)** ‡	23.5±26.6	22.2±27.8	23.3±26.8	0.493
**Hypertension**	837 (79.9)	211 (84.4)	1048 (80.8)	0.108
**Diabetes mellitus**	367 (35.1)	113 (45.2)	480 (37.0)	0.003
**Coronary artery disease**	228 (21.8)	69 (27.6)	297 (22.9)	0.049
**Peripheral vascular disease**	62 (5.9)	19 (7.6)	81 (6.2)	0.324
**Pulmonary embolism**	126 (12.0)	29 (11.6)	155 (12.0)	0.849
**Chronic liver disease**	29 (2.8)	12 (4.8)	41 (3.2)	0.099
**Respiratory disease**	120 (11.5)	31 (12.4)	151 (11.6)	0.678
**Chronic kidney disease**	279 (26.6)	88 (35.2)	367 (28.3)	0.007
**Atrial fibrillation**	436 (41.6)	92 (36.8)	528 (40.7)	0.161
**Deep vein thrombosis**	132 (12.6)	42 (16.8)	174 (13.4)	0.081
**Valve disease**	240 (22.9)	60 (24.0)	300 (23.1)	0.717
**Cerebrovascular attack**	480 (45.8)	101 (40.4)	581 (44.8)	0.120
**Thyroid disease**	68 (6.5)	12 (4.8)	80 (6.2)	0.317
**Malignancy**	213 (20.3)	58 (23.2)	271 (20.9)	0.318
**Congestive heart failure**	321 (30.7)	105 (42.0)	426 (32.8)	0.001

* Mean ± Standard deviation.

† The period from the administration of warfarin to the event of INR >3.0.

‡ The period from the administration of warfarin to the last visit or death of patients.

WRN, warfarin-related nephropathy; WFR, warfarin; INR, international normalized ratio; F/U, follow-up.

### Demographic and baseline clinical characteristics of patients

The demographic and baseline clinical characteristics classified by the development of WRN are shown in Table 1. Diabetes mellitus, coronary artery disease (CAD), CKD, and CHF were more frequently present in patients with WRN. However, there was no difference in male sex, mean age, other co-morbid diseases, indications for warfarin therapy, and follow-up period according to WRN.

### Baseline laboratory findings within 6 months before INR >3.0

Patients with WRN had lower basal Hb, Hct, serum calcium, total protein, and albumin levels than patients without WRN. On the other hand, there was no difference in basal INR, liver function test, sCr, and eGFR between the two groups (Table 2).

**Table 2:** Baseline laboratory findings of patients with and without WRN.

	No WRN (N = 1047, 80.7%)	WRN (N = 250, 19.3%)	Total (N = 1297)	*P*-value
**Prothrombin time (INR)**	1.67±0.57	1.65±0.55	1.66±0.56	0.632
**Serum creatinine (mg/dL)**	1.03±0.83	1.13±0.99	1.05±0.86	0.155
**MDRD-GFR (IDMS Cr) (ml/min)**	79.19±31.18	77.74±35.71	78.91±32.10	0.556
**Hemoglobin (g/dL)**	12.03±2.18	11.24±2.07	11.88±2.18	<0.001
**Hematocrit (%)**	36.25±6.28	33.92±6.34	35.80±6.36	<0.001
**Platelet (10^3^/ul)**	229.36±117.49	222.51±113.24	228.04±116.66	0.429
**Calcium (mg/dL)**	8.43±0.65	8.27±0.71	8.40±0.67	0.001
**Phosphorus (mg/dL)**	3.35±0.83	3.25±0.84	3.33±0.83	0.091
**Cholesterol (mg/dL)**	153.68±46.04	147.52±44.82	152.53±45.86	0.075
**Protein, total (g/dL)**	6.44±0.89	6.24±0.93	6.40±0.90	0.003
**Albumin (g/dL)**	3.63±0.57	3.43±0.61	3.59±0.58	<0.001
**Total bilirubin (mg/dL)**	1.09±1.22	1.31±2.18	1.13±1.45	0.155
**ALP (IU/L)**	90±54	100±64	92±56	0.011
**AST (GOT, IU/L)**	48±109	49±81	48±104	0.912
**ALT (GPT, IU/L)**	35±67	39±65	36±67	0.450

All values are described as “Mean ± Standard deviation”.

MDRD, modification of diet in renal disease; GFR, estimated glomerular filtration rate; IDMS, isotope dilution mass spectrometry; sCr, serum creatinine; ALP, alkaline phosphatase.

### Laboratory findings within 1 week after INR >3.0

**Figure 1:**
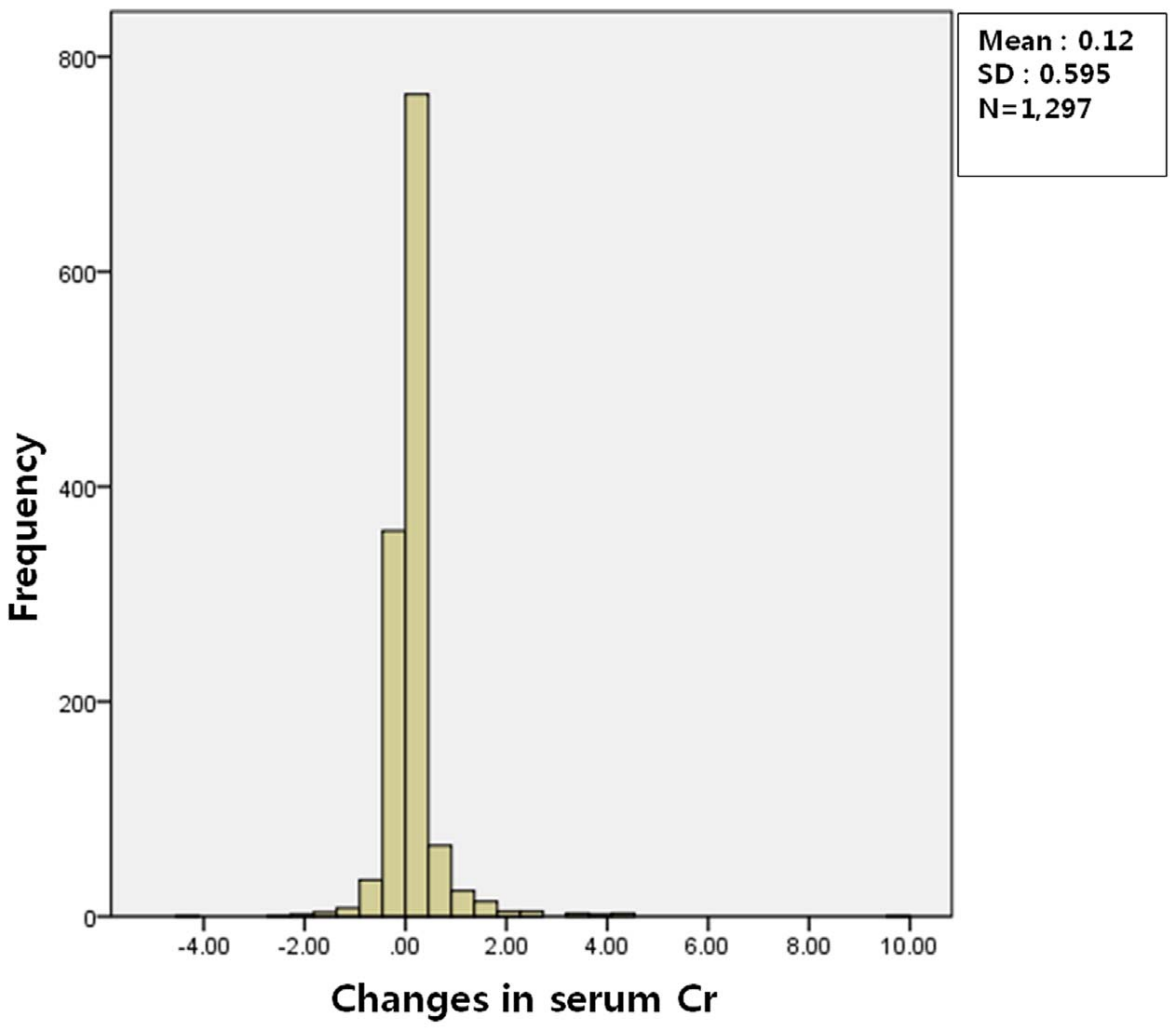
The distribution of the changes in serum creatinine from baseline to INR elevation. The histogram of the changes in serum creatinine from baseline value showed the normal distribution.

INR was higher in patients with WRN than patients without INR, and the increase in INR over basal INR was also higher in patients with WRN than without WRN. Although the Hb and Hct level were lower in patients with WRN than patients without WRN, there was no difference in the decrease in these values over basal Hb and Hct according to the presence of WRN, suggesting that there were no clinically significant bleeding events in our patients. Platelet counts in peripheral blood and the changes over basal platelet counts did not differ between the patients with and without WRN. The histogram of the changes in serum creatinine from baseline value showed the normal distribution (Figure 1). As expected from the definition of WRN, the sCr level was higher in patients with WRN than patients without WRN, and the percentages of increment of the sCr level and decrement of the eGFR over basal values were much greater in the WRN group. Liver function test assessed by serum AST and ALT was worse in WRN group (Table 3).

**Table 3:** Laboratory findings at the event of INR >3.0 according to presence of WRN.

	No WRN (N = 1047, 80.7%)	WRN (N = 250, 19.3%)	Total (N = 1297)	*P*-value
**Prothrombin time (INR)**	3.82±1.14	4.19±1.56	3.89±1.24	<0.001
**Δ PT (INR)**	2.15±1.25	2.54±1.63	2.23±1.34	<0.001
**Hemoglobin (g/dL)**	11.91±3.92	10.98±2.33	11.73±3.68	0.001
**Δ Hemoglobin (g/dL)**	−0.08±3.71	−0.30±1.74	−0.12±3.42	0.403
**Hematocrit (%)**	35.67±6.29	33.38±6.85	35.22±6.46	<0.001
**Δ Hematocrit (%)**	−0.49±4.71	−0.60±5.39	−0.51±4.85	0.781
**Platelet (103/ul)**	252.73±116.09	239.04 ±115.27	250.03±116.01	0.109
**Δ Platelet (103/ul)**	21.91±108.50	16.89±112.98	20.93±109.35	0.549
**Serum creatinine (mg/dL)**	0.98±0.66	1.93±1.44	1.16±0.94	<0.001
**ΔCreatinine (mg/dL)**	−0.05±0.29	0.81±0.94	0.12±0.59	<0.001
(ΔCr/base Cr)*100 (%)	−0.60±19.04	85.80±103.55	16.06±59.29	<0.001
**MDRD-GFR (IDMS Cr) (ml/min)**	81.83±40.01	42.06±20.77	74.16±40.27	<0.001
**Δ GFR (ml/min)**	2.64± 29.09	−35.69± 23.05	−4.75± 31.84	<0.001
**ALP (IU/L)**	99±68	119±88	102±72	0.001
**AST (GOT, IU/L)**	43±128	328±1110	95±501	<0.001
**ALT (GPT, IU/L)**	38±102	173±653	63±299	0.003

All values are described as “Mean ± Standard deviation”.

PT prothrombin time.

### Risk factors for the development of WRN

We analyzed risk factors for the development of WRN, as shown in Table S1 and Table 4. Of the co-morbid conditions, CHF (OR 1.65; 95% CI 1.23–2.21; p = 0.001) was the independent risk factors for the occurrence of WRN. In contrast, the presence of atrial fibrillation significantly decreased the risk for the development of WRN (Table 4). Although the risk for the occurrence of WRN increased along with the progression of the CKD stage in univariate analysis, this relationship was not valid in multivariate analysis. In addition, age and male gender were not associated with WRN (Table S1).

**Table 4:** Risk factors for development of WRN.

	Univariate Analysis		Multivariate Analysis*	
	OR (95% CI)	*P*-value	OR (95% CI)	*P*-value
**Atrial fibrillation**	0.58 (0.45–0.75)	<0.001	0.56 (0.40–0.80)	0.001
**Congestive heart failure**	1.49 (1.16–1.92)	0.002	1.64 (1.19–2.25)	0.002

Calcium (baseline)	1^st^ [6.0–7.9]	Reference		Reference	
	2^nd^ [8.0–8.4]	0.64 (0.46–0.88)	0.006	1.06 (0.72–1.57)	0.764
	3^rd^ [8.5–8.8]	0.30 (0.20–0.44)	<0.001	0.87 (0.35–0.94)	0.026
	4^th^ [8.9–10.9]	0.26 (0.18–0.38)	<0.001	0.64 (0.38–1.08)	0.092

Albumin (baseline)	1^st^ [1.1–3.1]	Reference		Reference	
	2^nd^ [3.2–3.6]	0.43 (0.31–0.61)	<0.001	0.46 (0.31–0.67)	<0.001
	3^rd^ [3.7–4.0]	0.26 (0.18–0.38)	<0.001	0.27 (0.18–0.42)	<0.001
	4^th^ [4.1–5.3]	0.15 (0.10–0.22)	<0.001	0.19 (0.12–0.30)	<0.001

AST (GOT) (at INR>3.0)	1^st^ [7]–[19]	Reference		Reference	
	2^nd^[20–25]	1.03 (0.67–1.57)	0.908	1.12 (0.70–1.79)	0.646
	3^rd^ [26–37]	1.12 (0.74–1.70)	0.595	1.43 (0.90–2.27)	0.130
	4^th^ [38–7002]	2.69 (1.86–3.88)	<0.001	2.29 (1.51–3.46)	<0.001

* Covariates: gender, age, comorbidities including diabetes mellitus, coronary artery disease, atrial fibrillation, deep vein thrombosis, congestive heart failure, and CKD stage, and laboratory findings including INR at baseline and at INR >3.0, baseline serum calcium, phosphorus, protein, albumin, cholesterol, and alkaline phosphatase level.

OR, odds ratio; CI, confidence interval.

Of the laboratory findings, lower basal level, including INR, serum calcium, phosphorus, protein, cholesterol, and alkaline phosphatase were correlated with the risk of WRN. However, after adjustment for other risk factors, these results were not found to be statistically significant. In addition, the INR level at the event of INR >3.0 did not influence the development of WRN (Table S1). In multivariate analysis after adjustment for age, gender, and statistically significant covariates in univariate analysis, the risk of WRN decreased as the basal serum albumin level increased [2^nd^ quartile (1.1–3.1) OR 0.50; 95% CI 0.34–0.74; p<0.001, 3^rd^ quartile (3.2–3.6) OR 0.34; 95% CI 0.21–0.54; p<0.001, 4^th^ quartile (4.1–5.3) OR 0.25; 95% CI 0.15–0.43; p<0.001] and increased in highest quartile serum AST level at post INR elevation [4^th^ quartile (38–7002) OR 2.29; 95% CI 1.51–3.46; p<0.001] (Table 4).

### Demographic and clinical characteristics of patients with and without atrial fibrillation

To exclude the possibility that observed protective effect of atrial fibrillation was related to benign clinical characteristics of patients with atrial fibrillation, we compared clinical characteristic according to the presence of atrial fibrillation. Patients with AF were older and had more frequent congestive heart failure which was independent risk factors for WRN in this study. In addition, co-morbidities such as hypertension, diabetes mellitus, respiratory disease, and cerebrovascular attack were more frequent in patient with AF. The patients without AF had more frequent thromboembolic events which might be related to the less aggressive anticoagulation in these patients as reflected by lower basal INR and INR, when INR exceed 3.0. These patients had higher frequency of malignancy (Table S2, S3, S4).

The patients with AF also had lower basal eGFR, although serum albumin level, another independent protective factor for WRN in this study was higher in these patients (Table S3). When INR exceeded 3.0, the patients with AF had lesser decline in eGFR than those without AF (Table S4). But renal functions after follow-up, which were assessed by eGFR, were still lower in patients with AF than without AF. (Table S5).

Although long-term mortality is higher in patients without AF, this might be related to higher frequency of malignancy in patients without AF. (Table S6).

### Frequency of the adverse events other than acute kidney injury according to the occurrence of WRN

To exclude the possibility that the severity of patient's condition rather than the warfarin-induced glomerular bleeding had influenced the decline in renal function in WRN group, we compared the frequency of the adverse events within 1 or 3 months after INR>3.0 between WRN and non-WRN group.

The acute illnesses include admission of any cause, acute illness newly diagnosed in patients, and visit to emergency room from any causes. (Table 5).

**Table 5:** The frequency of the adverse events according to the occurrence of WRN.

	No WRN (N = 1047, 80.7%)	WRN (N = 250, 19.3%)	*P*-value
**Admission rate within 1month (1)** †	19.5% (N = 204)	22.0% (N = 55)	0.379
**Admission rate within 3month (2)** ††	27.9% (N = 292)	33.6% (N = 84)	0.075
**Acute disease** ††† **within 1month (3)**	24.6% (N = 258)	28.0% (N = 70)	0.292
**Acute disease within 3month (4)**	35.4% (N = 371)	38.8% (N = 97)	0.341
**ER visit within 1month (5)**	21.4% (N = 224)	24.0% (N = 60)	0.395
**ER visit within 3month (6)**	28.4% (N = 297)	32.0% (N = 80)	0.278
**Sum of (1), (3), and (5)**	43.9% (N = 460)	45.6% (N = 114)	0.671
**Sum of (2), (4), and (6)**	57.0% (N = 597)	60.0% (N = 150)	0.433

† Post event (INR>3) 1 month.

†† Post event (INR>3) 3 month.

††† Acute disease includes infectious disease, ischemic heart disease, cerebrovascular disease, injuries, and poisoning.

There were no differences in frequency of the adverse events except slight higher rate of admission within 3 months in WRN group not supporting the major effect of patients, severe condition on decline in renal function in WRN group.

### Frequency of the use of non-nephrotoxic drugs according to the occurrence of WRN

As a another way to exclude the possibility that the severity of patient's condition rather than the warfarin-induced glomerular bleeding had influenced the decline in renal function in WRN group, we compared the frequency of prescription of non-nephrotoxic drugs which could be used to treat the acute illness between WRN and non-WRN group. (Table 6).

**Table 6:** The frequency of the use of non-nephrotoxic drugs according to the occurrence of WRN.

	No WRN (N = 1047, 80.7%)	WRN (N = 250, 19.3%)	*P*-value
**Prescription rate of drugs related to acute illness (%)**	98.9% (N = 1035)	99.6% (N = 249)	0.482

* Non-nephrotoxic drugs potentially associated with acute illness include antiplatelets, thrombolytics, inotropics, antibiotics, antiviral drug, antifungal drug, proton pump inhibitors, H2 blockers, analgesics, anesthetic drugs and so on.

There was no difference in prescription rate of therapeutic drugs between two groups, also not supporting the major effect of patients, severe condition on decline in renal function in WRN group.

### The impact of WRN on renal function after follow-up

The change in serum creatinine after follow-up from value within 1 week after INR>3.0 showed normal distribution (histogram not shown). Despite the similar basal renal function between the WRN and non-WRN groups, the sCr level was higher and the eGFR was lower in patients with WRN than those without WRN after follow-up. Interestingly, the INR level was still higher in patients with WRN than patients without WRN even after follow-up, although this finding barely reached statistical significance (Table 7). While there was no difference in renal function at post INR>3.0 and follow-up in non-WRN group according to the survival of patients, the renal function in dead patients was worse both post INR>3.0 and follow-up than live patients in WRN group (Table S7).

**Table 7:** The impact of WRN on renal function after follow-up.

	No WRN (N = 1047, 80.7%)	WRN (N = 250, 19.3%)	Total (N = 1297)	*P*-value
**Duration (months)** *	14.9±20.7	14.2±21.5	14.7±20.9	0.636
**PT (INR)**	2.35±1.53	2.57±1.80	2.39±1.59	0.074
**sCr (mg/dL)**	1.12±0.87	1.74±1.34	1.24±1.01	<0.001
**MDRD-GFR (ml/min)**	78.28±43.37	52.43±32.41	73.29±42.71	<0.001
**ΔCreatinine (mg/dL)**	0.14±0.69	−0.20±1.02	0.07±0.77	<0.001
**Δ GFR (ml/min)**	−3.46±42.56	10.37±26.70	−0.79±40.36	<0.001

All values are described as “Mean ± Standard deviation”.

* The period from the event of INR >3.0 to the last laboratory measurements.

### The impact of WRN on long-term mortality

**Figure 2:**
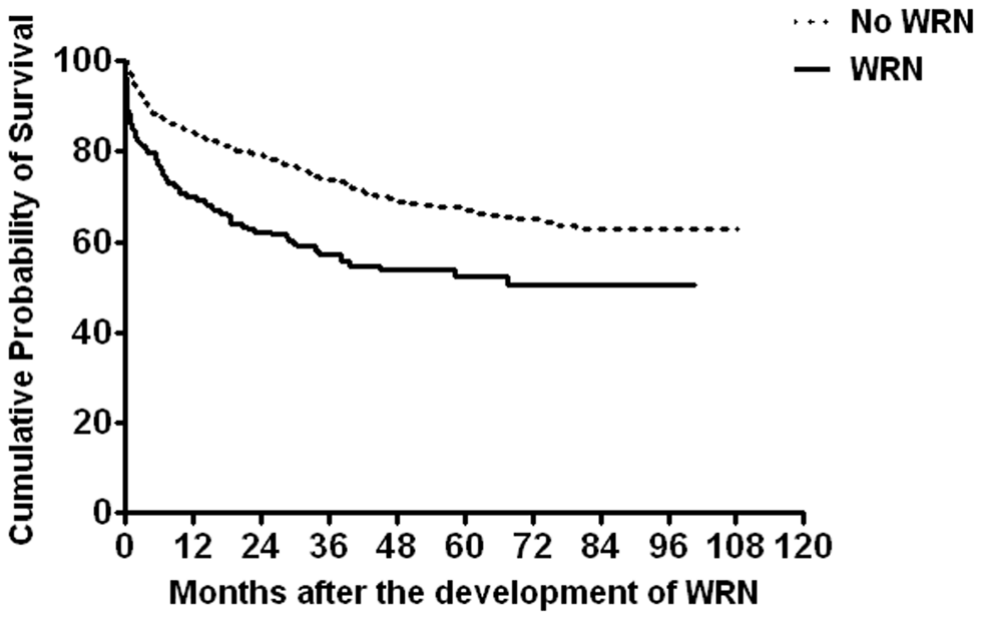
The impact of warfarin-related nephropathy on long-term mortality. Warfarin-related nephropathy significantly increased the mortality rate (p<0.001). The mortality rate was highest and survival difference between the two groups was greatest within 12 to 24 months after the occurrence of warfarin-related nephropathy, and decreased steadily and progressively over the time.

Long-term mortality according to WRN is demonstrated in Table 8 and Figure 2. The actual mortality rates were 42.8% in the WRN group, 26.3% in the non-WRN group, and 29.5% in all patients over follow-up periods for the groups that were similar in duration. The increased risk of death in patients with WRN compared to patients without WRN was highest during 2 years after INR >3.0, reaching 103.8% at 1 year and 91.9% at 2 years, and it sharply declined thereafter (50.6% at 5 years) (Table 8, Figure 2). The most common cause of death was the progression of underlying malignancy, followed by cerebrovascular and cardiovascular events in both patients with and without WRN (Table 9).

**Table 8:** The impact of WRN on long-term mortality.

	No WRN (N = 1047, 80.7%)	WRN (N = 250, 19.3%)	Total (N = 1297)	*P*-value
**Duration** * **(months)** †	32.6±26.4	25.6±26.1	31.2±26.4	<0.001
**Mortality rate (%)**	26.3 (N = 275)	42.8 (N = 107)	29.5 (N = 382)	
**12 months (%)**	15.9	32.4	19.1	
**24 months (%)**	20.9	40.1	24.5	
**60 months (%)**	32.8	49.4	36	

* Mean ± Standard deviation.

† The period from the event of INR >3.0 to the last visit or death of patients (from Statistics Korea).

**Table 9:** The causes of death.

Cause of death	No WRN (N, %)*	WRN (N, %)†	Total (N)	*P*-value
**Cardiovascular**	38 (13.8)	20 (18.7)	58	0.233
**Respiratory**	17 (6.2)	5 (4.7)	22	0.570
**Infection**	15 (5.5)	2 (1.9)	17	0.170
**Malignancy**	93 (33.8)	30 (28.0)	123	0.278
**Cerebrovascular**	62 (22.5)	18 (16.8)	80	0.217
**Others**	50 (18.2)	32 (29.9)	82	0.012
**Total**	275 (100)	107 (100)	382	

* Percentage of the cause of death among patients without WRN.

† Percentage of the cause of death among patients with WRN.

## Discussion

This study investigated the incidence, clinical features, risk factors, and renal and patient outcome of WRN in Korean patients who might have different genetic and environmental factors related to WRN than American patients in previous reports [3], [4], [6].

Similar to a previous report, WRN developed in 19.3% of patients who had excessive warfarinization, and the majority of cases of WRN occurred within 1 year after the initiation of warfarinization. While WRN also occurred more frequently in patients with CKD than those without CKD, the incidence of WRN in CKD patients was lower in our cohorts than in CKD patients in the previous report (24.0% vs. 33.0%) [3]. Moreover, CKD was not an independent risk factor for the development of WRN in multivariate analysis.

The reasons for these discrepancies are not clear but may be related to the following facts. First, in our study, CKD was defined solely by the level of eGFR, irrespective of the presence of hematuria or proteinuria, which may reflect glomerular damage prone to warfarin-induced glomerular bleeding. Recently, the comparison of effects of previous treatment regimens with and without warfarin on patients with IgA nephropathy suggested the detrimental effects of warfarin in patients who already sustained glomerular damage [10]. Secondly, basal levels of sCr and eGFR were not different between the WRN and non-WRN groups in our cohorts, contrary to the previous report, suggesting less severe nature of pre-existing renal damage in our patients with WRN.

The independent risk factors for the development of WRN in this study were coexisting CHF, low serum basal albumin level, and high serum AST level at post INR elevation. The mechanisms by which these risk factors increase the risk of WRN are not clear but seem to be related to higher INR after warfarinization. Since approximately 97% of warfarin becomes bound to plasma protein, primarily albumin, and the remaining 3% is the unbound fraction that exhibits pharmacologic effects and is metabolized and excreted from the body [11], hypoalbuminemia that results in a greater amount of the free form of warfarin may promote over-anticoagulation [12], [13]. Decreased metabolism of warfarin in the liver (combined with the reduction in production of coagulation factors in severe cases) and plasma volume expansion induced by CHF with resultant dilutional hypoalbuminemia may contribute to the development of WRN [14], [15].

We do not have any plausible explanations about why the presence of atrial fibrillation is protective for the development of WRN. The more severe clinical picture of patients with atrial fibrillation, such as old age, more comorbidities, and worse renal function at baseline and after follow-up than patients without atrial fibrillation denied the possibility that protective effect was due to the benign clinical characteristic of patients with atrial fibrillation.

Collectively, our findings suggest that caution is required in prescribing and monitoring warfarin treatment in patients with a low pre-treated serum albumin level, especially if patients have CHD, regardless of basal renal function.

Recently, there have been increasing concerns that AKI can lead to chronic kidney disease and can accelerate the progression of pre-existing chronic kidney disease [16]–[18]. Consistent with these notions, our study also demonstrated that after the mean follow-up period of 14 months, the sCr level was still higher and the eGFR remained lower in patients with WRN compared to those without WRN, despite no significant difference in the basal sCr level. The renal function after follow-up was also worse in dead patients than live patients only in WRN group.

Similar to the previous study [3], this study also found that WRN was associated with a substantial increase in the mortality rate, especially in the early period after WRN. However, contrary to the previous study, which showed no significant difference in mortality from the 6 months after WRN, increased mortality persisted even 5 years after WRN. The results of the present study correspond well with previous studies that reported that AKI was associated with an increased risk for long-term mortality after several years of follow-up [19], [20].

There were several limitations to the current study. First, our study was a retrospective study using only medical records; thus some essential information was often unavailable. Second, the criteria for determination of baseline laboratory findings were suboptimal. Any time within 6 months before INR >3.0 could become “baseline”, so the intervals from “baseline” laboratory findings to the event of INR >3.0 were inconsistent between enrolled patients. Third, the progression of underlying CKD during the “baseline” 6 months rather than WRN could be the cause of elevated sCr in some patients, although we excluded this possibility of progression of CKD by calculating the slope of the 1/sCr level using the sCr level measured at 12 months, 6 months, and 3 months before INR >3.0 in patients who had available data. Fourth, although we defined AKI based on AKIN criteria published in 2007 [9], the required time frame “elevation of sCr >0.3 mg/dL within 48 hours” could not be satisfied in all patients. Fifth, we could not analyze the association between WRN and hematuria, proteinuria, and albuminuria due to lots of missing data related to urinalysis. Sixth, our study could not provide any data to an important question about unique susceptibility of glomerular capillary to warfarin observed in this study i.e. how glomerular hemorrhage could occur alone, without other significant systemic bleedings? Finally, the most importantly the current study could not totally exclude the effect of acute illness rather than warfarin itself on the deterioration of renal function in WRN group, although we demonstrated no significant difference in the frequency of advers events and prescription of therapeutic drugs between WRN and non-WRN groups. This study did not consider several coexisting clinical factors that affect warfarin and albumin metabolism and renal function, including hypovolemia, sepsis, and medication concurrently prescribed with warfarin. These conditions may contribute to acute renal failure and cause deterioration in renal function, acting as confounding factors in the analysis of the effects of warfarin on renal function. Concurrent use of aspirin or an anti-platelet agent shown to play a major role in the development of WRN in previous studies was not examined. Accordingly, these limitations of our study require well-designed, prospective, and large cohort studies on WRN along with the studies related to the pathogenesis of WRN and biomarkers for diagnosis and prediction of WRN.

In conclusion, WRN developed in a considerable number of patients who had excessive warfarinization and in the early course of warfarin therapy in particular. It is noteworthy that irrespective of renal dysfunction, a lower basal serum albumin level, a higher serum AST level at post INR elevation, and comorbidity such as CHF were independent predictors of WRN. The long-term as well as short-term renal and patient outcomes were adversely affected by the occurrence of WRN in Korean patients.

## Supporting Information

Table S1 **Risk factors for development of WRN.**
(DOCX)

Table S2 **Demographic and clinical baseline characteristics according to presence of AF.**
(DOCX)

Table S3 **Baseline laboratory findings according to presence of AF.**
(DOCX)

Table S4 **Laboratory findings at the event of INR >3.0 according to presence of AF.**
(DOCX)

Table S5 **The impact of AF on renal function after follow-up.**
(DOCX)

Table S6 **The impact of AF on long-term mortality.**
(DOCX)

Table S7 **The comparison of serum creatinine and eGFR at baseline, post INR>3.0, and follow up between alive and dead patients according to presence of WRN.**
(DOCX)
