# Supplementary material for: The Occurrence of Warfarin-Related Nephropathy and Effects on Renal and Patient Outcomes in Korean Patients
Source: PLoS One. 2013 Apr 1;8(4):e57661. doi: 10.1371/journal.pone.0057661 (PMC3613349; doi:10.1371/journal.pone.0057661)
Supplement: Table S1 — Risk factors for development of WRN. (DOCX) [file pone.0057661.s001.docx]

**Table S1. Risk factors for development of WRN**

|  | | | | **Univariate Analysis** | | **Multivariate Analysis^*^** | |
| --- | --- | --- | --- | --- | --- | --- | --- |
|  | | | | **OR (95% CI)** | ***P*-value** | **OR (95% CI)** | ***P*-value** |
| **Male** | | | | 0.89 (0.70 – 1.15) | 0.374 |  |  |
| **Age** | | | | 1.01 (1.00 – 1.02) | 0.028 |  |  |
| **Hypertension** | | | | 1.18 (0.84 – 1.66) | 0.342 |  |  |
| **Diabetes mellitus** | | | | 1.26 (0.98 – 1.62) | 0.070 |  |  |
| **Coronary artery disease** | | | | 1.35 (1.02 – 1.78) | 0.035 |  |  |
| **Peripheral vascular disease** | | | | 1.39 (0.87 – 2.22) | 0.168 |  |  |
| **Chronic liver disease** | | | | 1.18 (0.66 – 2.10) | 0.586 |  |  |
| **Respiratory disease** | | | | 1.01 (0.69 – 1.46) | 0.980 |  |  |
| **Atrial fibrillation** | | | | 0.58 (0.45 – 0.75) | <0.001 | 0.56 (0.40 – 0.80) | 0.001 |
| **Deep vein thrombosis** | | | | 1.33 (0.95 – 1.85) | 0.097 |  |  |
| **Pulmonary embolism** | | | | 0.98 (0.66 – 1.44) | 0.915 |  |  |
| **Valvular disease** | | | | 1.22 (0.91 – 1.64) | 0.176 |  |  |
| **Cerebrovascular attack** | | | | 0.83 (0.65 – 1.07) | 0.160 |  |  |
| **Thyroid disease** | | | | 0.73 (0.41 – 1.30) | 0.279 |  |  |
| **Malignancy** | | | | 1.08 (0.80 – 1.44) | 0.632 |  |  |
| **Congestive heart failure** | | | | 1.49 (1.16 – 1.92) | 0.002 | 1.64 (1.19 – 2.25) | 0.002 |
| **Chronic kidney disease** | | Stage 1 - 2 | | Reference |  |  |  |
|  | | Stage 3 | | 1.33 (1.00-1.78) | 0.051 |  |  |
|  | | Stage 4 - 5 | | 2.59 (1.67-4.02) | <0.001 |  |  |
| **Prothrombin time (at INR > 3.0)** | 1^st^ [3.01 – 3.18] | | Reference | |  |  |  |
|  | 2^nd^ [3.19 – 3.44] | | 1.15 (0.79- 1.68) | | 0.459 |  |  |
|  | 3^rd^ [3.45 – 3.98] | | 1.09 (0.74- 1.61) | | 0.653 |  |  |
|  | 4^th^ [3.99 – 12.65] | | 1.41 (1.00- 2.00) | | 0.053 |  |  |
| **Prothrombin time (baseline)** | 1^st^ [0.80 – 1.18] | | Reference | |  |  |  |
|  | 2^nd^ [1.19 – 1.47] | | 0.89 (0.63- 1.27) | | 0.527 |  |  |
|  | 3^rd^ [1.48 – 2.09] | | 0.69 (0.49-0.98) | | 0.040 |  |  |
|  | 4^th^ [2.10 – 2.99] | | 0.63 (0.44- 0.90) | | 0.010 |  |  |
| **Serum calcium (baseline)** | 1^st^ [6.0 – 7.9] | | Reference | |  | Reference |  |
|  | 2^nd^ [8.0 – 8.4] | | 0.64 (0.46-0.88) | | 0.006 | 1.06 (0.72-1.57) | 0.764 |
|  | 3^rd^ [8.5 – 8.8] | | 0.30 (0.20-0.44) | | <0.001 | 0.87 (0.35-0.94) | 0.026 |
|  | 4^th^ [8.9 – 10.9] | | 0.26 (0.18-0.38) | | <0.001 | 0.64 (0.38-1.08) | 0.092 |
| **Serum phosphorus (baseline)** | 1^st^ [0.9 – 2.8] | | Reference | |  |  |  |
|  | 2^nd^ [2.9 – 3.2] | | 0.65 (0.45 - 0.93) | | 0.017 |  |  |
|  | 3^rd^ [3.3 – 3.7] | | 0.65 (0.47 - 0.91) | | 0.012 |  |  |
|  | 4^th^ [3.8 – 9.9] | | 0.63 (0.44 - 0.90) | | 0.012 |  |  |
| **Serum protein (baseline)** | 1^st^[3.5 – 5.8] | | Reference | |  |  |  |
|  | 2^nd^ [5.9 – 6.4] | | 0.40 (0.28 - 0.57) | | <0.001 |  |  |
|  | 3^rd^ [6.5 – 7.0] | | 0.33 (0.23 - 0.47) | | <0.001 |  |  |
|  | 4^th^[7.1 – 8.7] | | 0.21 (0.14 - 0.31) | | <0.001 |  |  |
| **Serum albumin (baseline)** | 1^st^ [1.1 – 3.1] | | Reference | |  | Reference |  |
|  | 2^nd^ [3.2 – 3.6] | | 0.43 (0.31 - 0.61) | | <0.001 | 0.46 (0.31- 0.67) | <0.001 |
|  | 3^rd^ [3.7 – 4.0] | | 0.26 (0.18 - 0.38) | | <0.001 | 0.27 (0.18-0.42) | <0.001 |
|  | 4^th^ [4.1 – 5.3] | | 0.15 (0.10 - 0.22) | | <0.001 | 0.19 (0.12-0.30) | <0.001 |
| **Serum cholesterol (baseline)** | 1^st^ [35 – 118] | | Reference | |  |  |  |
|  | 2^nd^[119 – 151] | | 0.72 (0.49-1.04) | | 0.082 |  |  |
|  | 3^rd^ [152 – 182] | | 0.58 (0.40- 0.83) | | 0.003 |  |  |
|  | 4^th^ [183 – 334] | | 0.39 (0.26-0.59) | | <0.001 |  |  |
| **ALP**  **(baseline)** | 1^st^ [20 – 61] | | Reference | |  |  |  |
|  | 2^nd^[62 – 78] | | 1.06 (0.69-1.61) | | 0.792 |  |  |
|  | 3^rd^ [79 – 102] | | 1.61 (1.09- 2.38) | | 0.017 |  |  |
|  | 4^th^ [103 – 588] | | 2.03 (1.38-2.99) | | <0.001 |  |  |
| **AST (GOT)**  **(at INR>3.0)** | 1^st^ [7 – 19] | | Reference | |  | Reference |  |
|  | 2^nd^[20 – 25] | | 1.03 (0.67-1.57) | | 0.908 | 1.12 (0.70- 1.79) | 0.646 |
|  | 3^rd^ [26 – 37] | | 1.12 (0.74- 1.70) | | 0.595 | 1.43 (0.90-2.27) | 0.130 |
|  | 4^th^ [38 – 7002] | | 2.69 (1.86-3.88) | | <0.001 | 2.29 (1.51-3.46) | <0.001 |
| **ALT (GPT)**  **(at INR>3.0)** | 1^st^ [3 – 14] | | Reference | |  |  |  |
|  | 2^nd^[15 – 21] | | 0.74 (0.50-1.10) | | 0.142 |  |  |
|  | 3^rd^ [22 – 36] | | 0.86 (0.58- 1.27) | | 0.447 |  |  |
|  | 4^th^ [37 – 6800] | | 1.62 (1.13-2.31) | | 0.008 |  |  |

^*^ Covariates: gender, age, comorbidities including diabetes mellitus, coronary artery disease, atrial fibrillation, deep vein thrombosis, congestive heart failure, and CKD stage, and laboratory findings including INR at baseline and at INR > 3.0, baseline serum calcium, phosphorus, protein, albumin, cholesterol, and alkaline phosphatase level.

OR, odds ratio; CI, confidence interval
