# Supplementary material for: The Occurrence of Warfarin-Related Nephropathy and Effects on Renal and Patient Outcomes in Korean Patients
Source: PLoS One. 2013 Apr 1;8(4):e57661. doi: 10.1371/journal.pone.0057661 (PMC3613349; doi:10.1371/journal.pone.0057661)
Supplement: Table S2 — Demographic and clinical baseline characteristics according to presence of AF. (DOCX) [file pone.0057661.s002.docx]

**Table S2. Demographic and clinical baseline characteristics according to presence of AF**

|  | **With AF (N=528, 40.7%)** | **Without AF (N=769, 59.3%)** | ***P*-value** |
| --- | --- | --- | --- |
| **Male (%)** | 54.0 | 50.1 | 0.175 |
| **Age^*^** | 71.1 ± 10.0 | 66.5 ± 13.7 | <0.001 |
| **Duration^*^ (WFR-INR > 3.0)^†^** | 13.5 ± 20.9 | 5.1 ± 12.7 | <0.001 |
| **Duration^*^ (WFR-F/U)^‡^** | 31.2 ± 29.3 | 17.8 ± 23.4 | <0.001 |
| **Hypertension (%)** | 90.5 | 74.1 | <0.001 |
| **Diabetes mellitus (%)** | 41.5 | 33.9 | 0.006 |
| **Coronary artery disease (%)** | 22.7 | 23.0 | 0.946 |
| **Peripheral vascular disease (%)** | 5.1 | 7.0 | 0.199 |
| **Pulmonary embolism (%)** | 2.5 | 18.5 | <0.001 |
| **Chronic liver disease (%)** | 4.0 | 2.6 | 0.196 |
| **Respiratory disease (%)** | 13.8 | 10.1 | 0.043 |
| **Chronic kidney disease (%)** | 30.9 | 26.5 | 0.091 |
| **Deep vein thrombosis (%)** | 4.9 | 19.2 | <0.001 |
| **Valve disease (%)** | 25.0 | 21.8 | 0.203 |
| **Cerebrovascular attack (%)** | 54.0 | 38.5 | <0.001 |
| **Thyroid disease (%)** | 8.1 | 4.8 | 0.018 |
| **Malignancy (%)** | 15.0 | 25.0 | <0.001 |
| **Congestive heart failure (%)** | 42.2 | 26.4 | <0.001 |

^*^Mean ± Standard deviation

**^†^**The period from the administration of warfarin to the event of INR > 3.0

**^‡^**The period from the administration of warfarin to the last visit or death of patients
