# Supplementary material for: The Occurrence of Warfarin-Related Nephropathy and Effects on Renal and Patient Outcomes in Korean Patients
Source: PLoS One. 2013 Apr 1;8(4):e57661. doi: 10.1371/journal.pone.0057661 (PMC3613349; doi:10.1371/journal.pone.0057661)
Supplement: Table S3 — Baseline laboratory findings according to presence of AF. (DOCX) [file pone.0057661.s003.docx]

**Table S3. Baseline laboratory findings according to presence of AF**

|  | **With AF (N=528, 40.7%)** | **Without AF (N=769, 59.3%)** | ***P*-value** |
| --- | --- | --- | --- |
| **Prothrombin time (INR)** | 1.75 ± 0.59 | 1.60 ± 0.54 | <0.001 |
| **Serum creatinine (mg/dL)** | 1.05 ± 0.70 | 1.05 ± 0.96 | 0.971 |
| **MDRD-GFR (IDMS Cr) (ml/min)** | 74.4 ± 28.3 | 82.0 ± 34.1 | <0.001 |
| **Hemoglobin (g/dL)** | 12.7 ± 2.2 | 11.4 ± 2.0 | <0.001 |
| **Hematocrit (%)** | 37.23 ± 6.51 | 34.03 ± 6.13 | <0.001 |
| **Platelet (10^3^/ul)** | 217.0 ± 94.1 | 235.1 ± 128.6 | 0.006 |
| **Calcium (mg/dL)** | 8.6 ± 0.6 | 8.3 ± 0.7 | <0.001 |
| **Phosphorus (mg/dL)** | 3.3 ± 0.8 | 3.3 ± 0.9 | 0.633 |
| **Cholesterol (mg/dL)** | 157 ± 42 | 150 ± 48 | 0.010 |
| **Protein, total (g/dL)** | 6.6 ± 0.9 | 6.2 ± 0.9 | <0.001 |
| **Albumin (g/dL)** | 3.8 ± 0.5 | 3.5 ± 0.6 | <0.001 |
| **Total bilirubin (mg/dL)** | 1.2 ± 1.5 | 1.1 ± 1.4 | 0.263 |

All values are described as “Mean ± Standard deviation”.
