# Supplementary material for: The Occurrence of Warfarin-Related Nephropathy and Effects on Renal and Patient Outcomes in Korean Patients
Source: PLoS One. 2013 Apr 1;8(4):e57661. doi: 10.1371/journal.pone.0057661 (PMC3613349; doi:10.1371/journal.pone.0057661)
Supplement: Table S4 — Laboratory findings at the event of INR >3.0 according to presence of AF. (DOCX) [file pone.0057661.s004.docx]

**Table S4. Laboratory findings at the event of INR > 3.0 according to presence of AF**

|  | **With AF (N=528, 40.7%)** | **Without AF (N=769, 59.3%)** | ***P*-value** |
| --- | --- | --- | --- |
| **Prothrombin time (INR)** | 3.93 ± 1.27 | 3.86 ± 1.21 | 0.296 |
| **Δ PT (INR)** | 2.18 ± 1.37 | 2.26 ± 1.31 | 0.305 |
| **Hemoglobin (g/dL)** | 12.4 ± 2.3 | 11.4 ± 4.3 | <0.001 |
| **Δ Hemoglobin (g/dL)** | 0.32 ± 1.61 | 0.01 ± 4.11 | 0.158 |
| **Hematocrit (%)** | 37.23 ± 6.51 | 34.03 ± 6.13 | <0.001 |
| **Δ Hematocrit (%)** | 0.75 ± 4.75 | 0.37 ± 4.90 | 0.214 |
| **Platelet (10^3^/ul)** | 245.2 ± 106.6 | 252.9 ± 121.2 | 0.261 |
| **Δ Platelet (10^3^/ul)** | -27.7 ± 96.2 | -17.0 ± 116.2 | 0.122 |
| **Serum creatinine (mg/dL)** | 1.15 ± 0.78 | 1.18 ± 1.04 | 0.545 |
| **ΔCreatinine (mg/dL)** | 0.10 ± 0.41 | 0.13 ± 0.69 | 0.294 |
| **(ΔCr/base Cr)*100 (%)** | 12.8 ± 44.8 | 18.3 ± 67.4 | 0.075 |
| **MDRD-GFR (IDMS Cr) (ml/min)** | 71.85 ± 44.94 | 75.75 ± 36.66 | 0.086 |
| **Δ GFR (ml/min)** | -2.69 ± 37.09 | -6.40 ± 27.63 | 0.039 |

All values are described as “Mean ± Standard deviation”.
