# Supplementary material for: The Occurrence of Warfarin-Related Nephropathy and Effects on Renal and Patient Outcomes in Korean Patients
Source: PLoS One. 2013 Apr 1;8(4):e57661. doi: 10.1371/journal.pone.0057661 (PMC3613349; doi:10.1371/journal.pone.0057661)
Supplement: Table S5 — The impact of AF on renal function after follow-up. (DOCX) [file pone.0057661.s005.docx]

**Table S5. The impact of AF on renal function after follow-up**

|  | **With AF (N=528, 40.7%)** | **Without AF (N=769, 59.3%)** | ***P*-value** |
| --- | --- | --- | --- |
| **Duration (months)^*^** | 17.7 ± 22.2 | 12.7 ± 19.6 | <0.001 |
| **PT (INR)** | 2.48 ± 1.70 | 2.34 ± 1.50 | 0.103 |
| **sCr (mg/dL)** | 1.22 ± 0.88 | 1.25 ± 1.09 | 0.604 |
| **MDRD-GFR (ml/min)** | 68.61 ± 34.45 | 76.65 ± 47.24 | <0.001 |
| **ΔCreatinine (mg/dL)** | 0.07 ± 0.62 | 0.07 ± 0.87 | 0.956 |
| **Δ GFR (ml/min)** | -3.24 ± 40.85 | 0.89 ± 39.95 | 0.070 |

All values are described as “Mean ± Standard deviation”.

^*^The period from the event of INR > 3.0 to the last laboratory measurements
