# Supplementary material for: The Occurrence of Warfarin-Related Nephropathy and Effects on Renal and Patient Outcomes in Korean Patients
Source: PLoS One. 2013 Apr 1;8(4):e57661. doi: 10.1371/journal.pone.0057661 (PMC3613349; doi:10.1371/journal.pone.0057661)
Supplement: Table S6 — The impact of AF on long-term mortality. (DOCX) [file pone.0057661.s006.docx]

**Table S6. The impact of AF on long-term mortality**

|  | **With AF (N=528, 40.7%)** | **Without AF (N=769, 59.3%)** | ***P*-value** |
| --- | --- | --- | --- |
| **Duration^*^(months)^†^** | 32.3 ± 26.4 | 30.5 ± 26.5 | 0.227 |
| **Mortality rate (%)** | 25.4 | 32.2 | 0.008 |
| **Causes of death** |  |  |  |
| **Cancer** | 14.9 | 41.5 |  |
| **Cerebrovascular** | 27.6 | 17.3 |  |
| **Cardiovascular** | 20.9 | 12.1 |  |

**^*^** Mean ± Standard deviation

**^†^** The period from the event of INR > 3.0 to the last visit or death of patients (from Statistics Korea)

**.**
