# Supplementary material for: The Occurrence of Warfarin-Related Nephropathy and Effects on Renal and Patient Outcomes in Korean Patients
Source: PLoS One. 2013 Apr 1;8(4):e57661. doi: 10.1371/journal.pone.0057661 (PMC3613349; doi:10.1371/journal.pone.0057661)
Supplement: Table S7 — The comparison of serum creatinine and eGFR at baseline, post INR>3.0, and follow up between alive and dead patients according to presence of WRN. (DOCX) [file pone.0057661.s007.docx]

**Table S7. The comparison of serum creatinine and eGFR at baseline, post INR>3.0, and follow up between alive and dead patients according to presence of WRN**

|  |  | **Alive (N=772, 73.7%)** | **Dead (N=275, 26.3%)** | ***P*-value** |
| --- | --- | --- | --- | --- |
| **No WRN group** | **Baseline-sCr (mg/dL)** | 1.02 ± 0.85 | 1.05 ± 0.75 | 0.574 |
|  | **Baseline-MDRD-GFR (ml/min)** | 79.0 ± 30.5 | 79.7 ± 33.0 | 0.734 |
|  | **INR>3-sCr (mg/dL)** | 0.97 ± 0.67 | 1.00 ± 0.61 | 0.646 |
|  | **INR>3-MDRD-GFR (ml/min)** | 81.6 ± 41.2 | 82.4 ± 36.5 | 0.768 |
|  | **F/U-sCr (mg/dL)** | 1.05 ± 0.77 | 1.29 ± 1.08 | 0.001 |
|  | **F/U-MDRD-GFR (ml/min)** | 77.0 ± 32.4 | 82.2 ± 64.7 | 0.204 |
| **WRN group** | **Baseline-sCr (mg/dL)** | 1.08 ± 0.60 | 1.18 ± 1.35 | 0.437 |
|  | **Baseline-MDRD-GFR (ml/min)** | 77.2 ± 35.4 | 78.4 ± 36.2 | 0.801 |
|  | **INR>3-sCr (mg/dL)** | 1.74 ± 1.08 | 2.19 ± 1.78 | 0.023 |
|  | **INR>3-MDRD-GFR (ml/min)** | 44.9 ± 20.2 | 38.3 ± 21.0 | 0.013 |
|  | **F/U-sCr (mg/dL)** | 1.47 ± 1.07 | 2.10 ± 1.56 | <0.001 |
|  | **F/U-MDRD-GFR (ml/min)** | 59.7 ± 32.3 | 42.8 ± 30.0 | <0.001 |

Note that follow-up eGFR was higher despite of higher serum Cr in dead patients. This may be related to the difference of other factors in MDRD equation. Also standard deviation was very wide in this group.
